# Molecular Analysis of the *SRD5A1* and *SRD5A2* Genes in Patients with Benign Prostatic Hyperplasia with Regard to Metabolic Parameters and Selected Hormone Levels

**DOI:** 10.3390/ijerph14111318

**Published:** 2017-10-30

**Authors:** Aleksandra Rył, Iwona Rotter, Anna Grzywacz, Iwona Małecka, Karolina Skonieczna-Żydecka, Katarzyna Grzesiak, Marcin Słojewski, Aleksandra Szylińska, Olimpia Sipak-Szmigiel, Małgorzata Piasecka, Kinga Walczakiewicz, Maria Laszczyńska

**Affiliations:** 1 Department of Histology and Developmental Biology, Pomeranian Medical University, 70-210 Szczecin, Poland; aleksandra.ryl@pum.edu.pl (A.R.); kasia.grzesiak302@gmail.com (K.G.); mpiasecka@ipartner.com.pl (M.P.); walczakiewicz.kinga@wp.pl (K.W.); maria@laszczynska.pl (M.L.); 2 Department of Medical Rehabilitation and Clinical Physiotherapy, Pomeranian Medical University, 70-210 Szczecin, Poland; iwona.rotter@pum.edu.pl; 3 Independent Laboratory of Health Promotion, Pomeranian Medical University, 70-103 Szczecin, Poland; annagrzywacz@gazeta.pl; 4 Department of Psychiatry, Pomeranian Medical University, 71-460 Szczecin, Poland; i.malecka@wp.pl; 5 Department of Biochemistry and Human Nutrition, Pomeranian Medical University, 71-460 Szczecin, Poland; karzyd@pum.edu.pl; 6 Department of Urology and Urological Oncology, Pomeranian Medical University, 70-111 Szczecin, Poland; mslojewski@gmail.com; 7 Department of Obstetrics and Gynecology, Pomeranian Medical University, 70-111 Szczecin, Poland; olimpiasipak-szmigiel@wp.pl

**Keywords:** benign prostatic hyperplasia, *SRD5A1* and *SRD5A2* genes, metabolic disorders, hormones

## Abstract

*Introduction*: The etiology of benign prostatic hyperplasia (BPH) has not so far been fully explicated. However, it is assumed that changes in the levels of hormones associated with aging can contribute to the development of prostatic hyperplasia. Dihydrotestosterone combines with the androgen receptor (AR) proteins of the prostate gland. Enzyme activity is based on two isoenzymes: type 1 and type 2. 5α-reductase type 1 is encoded by the *SRD5A1* gene, and type 2 is encoded by the *SRD5A2* gene. The aim of our study was to determine the frequency of the *SRD5A1* (rs6884552, rs3797177) and *SRD5A2* (rs523349, rs12470143) genes’ polymorphisms, and to assess the relationships between the genotypes of the tested mutations, and the levels of biochemical and hormonal parameters in patients with BPH. *Material and Methods*: The study involved 299 men with benign prostatic hyperplasia. We determined the serum levels of particular biochemical parameters—fasting plasma glucose (FPG), total cholesterol (TC), low-density lipoprotein (LDL), high-density lipoprotein (HDL) and triglycerides (TG)—by the spectrophotometric method, using ready reagent kits. The ELISA method was used to determine the levels of the following hormonal parameters and proteins: total testosterone (TT), free testosterone (FT), insulin (I), luteinizing hormone (LH), and sex hormone binding protein (SHBG). Metabolic syndrome was diagnosed. Genotyping was performed by real-time PCR. *Results*: We analyzed the relationships between the incidence of particular diseases and the genotypes of the *SRD5A1* and *SRD5A2* polymorphisms among patients with BPH. The BPH patients with the CC genotype of the *SRD5A2* rs523349 and rs12470143 polymorphisms were considerably less frequently diagnosed with metabolic syndrome (MetS) (*p* = 0.022 and *p* = 0.023 respectively). Our analysis revealed that homozygotes with the CC of the *SDR5A2* rs12470143 polymorphism had visibly higher HDL levels than those with the TT and CT genotypes (*p* = 0.001). Additionally, we found that the patients with the CC genotype of the *SDR5A2* rs12470143 polymorphism had considerably higher FT levels (*p* = 0.001) than the heterozygotes with the CT and the homozygotes with the TT of the genetic variant analyzed in our study. Furthermore, the patients with at least one G allele of the *SRD5A2* rs523349 polymorphism had significantly lower SGBG levels (*p* = 0.022) compared with the homozygotes with the CC genotype. The presence of at least one A allele (AA + AG genotypes) of the *SRD5A1* rs3797177 polymorphism entailed notably lower serum insulin levels than those observed in homozygotes with the GG genotype (*p* = 0.033). *Conclusions*: The study described in this article shows that selected *SRD5A1* and *SRD5A2* polymorphisms can alter the levels of metabolic and hormonal parameters in patients with BPH. Special attention should be paid to the *SDR5A2* rs12470143 polymorphism, which is associated with a change in lipid profile, as well as with the inheritance and incidence rate of MetS among these patients. An analysis of the frequency of this polymorphism among BPH patients could be useful in estimating the risk of getting ill, and planning therapies of concomitant diseases for BPH patients.

## 1. Introduction

Benign prostatic hyperplasia (BPH) is a serious social issue. As the estimates show, histopathological changes in prostate cell proliferation are observed in more than 50% of men over 50 years old, and their incidence rate increases with age. Due to lengthening life expectancy, the number of men affected by this problem will be growing [1].

The etiology of BPH has not so far been fully explicated. However, it is assumed that changes in the levels of hormones associated with aging can contribute to the development of prostatic hyperplasia. In men over 50, the blood testosterone level starts to decline, which leads to an increase in the level of dihydrotestosterone (DHT) produced from testosterone by the enzyme 5α-reductase [2].

Dihydrotestosterone combines with the androgen receptor (AR) proteins of the prostate gland. The resultant DHT–AR complex stimulates the proliferation of prostate cells, causing prostatic hyperplasia. Enzyme activity is based on two isoenzymes: type 1 and type 2 [3]. 5α-reductase type 1 is encoded by the *SRD5A1* gene located on the short arm of chromosome 5, and is mostly found in cell microsomes. The gene for 5α-reductase type 2 (*SRD5A2*) is located on the short arm of chromosome 2 [4]. This isoenzyme is present in both nuclear and microsomal fractions. Both types of 5α-reductase can occur in almost the whole organism, with particular isotypes prevailing in various tissues and organs. Apart from reproductive organs, type 1 is observed in the skin, liver, epidermis, sebaceous and sweat glands, hair follicles, endothelial cells of minor blood vessels, and Schwann cells of myelinated dermal nerves [5]. Its deficiency does not cause any disruption of sex differentiation [6]. 5α-reductase type 2 is characteristic of the prostate, skin in the reproductive organ area, sebaceous gland canals, and hair follicles. A deficiency of 5α-reductase type 2 results in male sexual differentiation disorders [6,7].

There are two types of 5α-reductase in a prostate gland with BPH. Both type 1 and type 2 can be found in prostate epithelial cells, but type 2 is also observed in prostate stromal cells. The available literature provides few publications concerning the influence of genetic variations in the *SDR5A1* and *SRD5A2* genes on the prostate function and size in the course of BPH [8,9,10]. Selected polymorphic variants of the *SDR5A1* and *SRD5A2* genes can influence the activity of hormones that are essential for BPH etiology, thus contributing to individual responsiveness to treatment [11].

The aim of our study was to determine the frequency of the *SRD5A1* (rs6884552, rs3797177) and *SRD5A2* (rs523349, rs12470143) genes’ polymorphisms, and to assess the relationships between the genotypes of the tested mutations and the levels of biochemical and hormonal parameters in patients with BPH.

## 2. Materials and Methods

The study involved 299 men aged 44–85 years (the mean age ± SD: 67.04 ± 7.50). They were patients admitted to the Clinic of Urology and Urologic Oncology at Pomeranian Medical University in Szczecin for planned transurethral resection of the prostate (TURP). The criteria for exclusion from the study were cancerous disease, active alcoholic disease, liver and thyroid conditions, as well as treatment with steroid or neuroleptic agents. BPH patients were treated with 5α-reductase inhibitor (oral finasteride in daily doses of 5 mg). The participants were informed about the course and purpose of the study, and gave their written consent to take part in it. The study was conducted with the consent of the Bioethical Commission of Pomeranian Medical University in Szczecin (permission number KB-0012/132/12) and was carried out in accordance with the Declaration of Helsinki. The research was financed by: FSN-322-08/14 and WNoZ-322- 03/S/2016.

### 2.1. Clinical Examination

Anthropometric data (weight, height, age, waist size, and blood pressure) were obtained from the patients. The participants were also asked to answer demographics questions. Body Mass Index (BMI) was calculated; we assumed that BMI in the range of 18.5–24.99 denoted normal weight, 25–29.99 indicated overweight, and of ≥30 meant obesity.

Metabolic syndrome was diagnosed according to the International Diabetes Federation (IDF) criteria of 2005 (abdominal circumference ≥94 cm, and at least two of the following deviations: fasting plasma glucose ≥100 mg/dL or treatment for type 2 diabetes, arterial blood pressure ≥130/85 mmHg or treatment for hypertension, the level of HDL cholesterol <40 mg/dL in men or treatment for dyslipidemia, the levels of triglycerides (TG) ≥150 mg/dL or treatment for dyslipidemia) [12].

### 2.2. Laboratory Analysis

Blood was taken from the tested men on an empty stomach from an ulnar vein between 7:30 a.m. and 9:00 a.m. For the biochemical and hormonal assays, blood was drawn into a tube with a coagulator and gel separator, and then centrifuged. For the genetic assays, blood was collected into tubes with EDTA (anticoagulant). The sera were stored at −70 °C.

We determined serum levels of biochemical parameters―fasting plasma glucose (FPG) in the non-diabetic men only, total cholesterol (TC), low-density lipoprotein (LDL), high-density lipoprotein (HDL), and triglycerides (TG)―by the spectrophotometric method, using ready reagent kits (Biolabo, Aqua-Med, Łódź, Poland). The ELISA method was used to determine the levels of the following hormonal parameters and proteins: total testosterone (TT), free testosterone (FT), insulin (I), luteinizing hormone (LH), and sex hormone binding protein (SHBG).

### 2.3. Data Collection

Genomic DNA from peripheral blood leukocytes was extracted using a High Pure Polymerase Chain Reaction (PCR) Template Preparation extraction kit (Roche Diagnostics, Mannheim, Germany). The extraction was performed according to the manufacturer’s instructions. DNA samples were stored at 4 °C for further analysis.

### 2.4. Genotyping of the SRD5A1 and SRD5A1 Genes

All of the laboratory procedures were carried out blind to diagnostic assessment. Genotyping was performed by real-time PCR using the Light Cycler System 2.0 (Roche Diagnostic, Basel, Switzerland). For the polymorphisms within the *SRD5A1* (rs = 6884552, rs = 3797177) and *SRD5A1* (rs = 523349, rs = 12470143) genes, 50 ng DNA in a total volume of 20 mL containing 2 mL reaction mix, 0.5 mM of each primer, 0.2 mM of each hybridization probe and 2 mM MgCl_2_ were used. The following PCR conditions were applied: 35 cycles of denaturation (95 °C for 10 min), annealing (60 °C for 10 s), and extension (72 °C for 15 s). After amplification, a melting curve was generated by holding the reaction at 40 °C for 20 s, and then heating slowly to 85 °C. The fluorescence signal was plotted against temperature to give melting curves for each sample.

For each *loci*, we performed analyses in overdominant models of inheritance, followed by recessive models of inheritance (*SRD5A1*: rs6884552 TT + TC vs. CC; rs3797177 AA + AG vs. GG and *SRD5A2*: rs523349 GG + CG vs. CC; rs12470143 TT + CT vs. CC).

### 2.5. Haplotype Analysis 

Analysis of the frequency of the tested Single Nucleotide Polymorphism (SNP) haplotypes was performed using the R haplo.stats package, version 1.2 (GNU Project, International Corporation, New York, NY, USA).

### 2.6. Statistical Analysis

Statistical analysis was performed with Statistica 12 software (StatSoft, Inc., Tulsa, OK, USA). Continuous variables were characterized by arithmetic mean (X) with standard deviation (SD), median (Med), and range. In the description of qualitative variables, we presented the number (*n*), which was also expressed as a percentage (%). A χ^2^ test was used to verify that genotype frequencies fit to the Hardy–Weinberg equilibrium (H–W). The Kruskalla–Willisa test was used to assess the associations between the genotypes *SRD5A1* (rs = 6884552, rs = 3797177) and *SRD5A1* (rs = 523349, rs = 12470143), polymorphisms and the anthropometric indicators, and hormonal and metabolic parameters. The non-parametric Mann–Whitney and parametric t-Student tests were employed to compare variables between genotype groups.

The assessment of relationships between genotypes and the qualitative variables was performed with a χ^2^ test of independence. For determination of the odds ratio (OR) and 95% confidence intervals (95% CI), we used logistic regression. The level of significance was set at *p* ≤ 0.05. For each of the *loci*, we performed analyses in overdominant models of inheritance, followed by adopted recessive models of inheritance; *SRD5A1*: TT + TC vs. CC (rs68845520), AA + AG vs. GG (rs = rs3797177), *SRD5A2*: GG + CG vs. CC (rs523349), TT + CT vs. CC (rs12470143).

## 3. Results

The study involved 299 men with BPH. Table 1 shows their characteristics. Overweight was diagnosed in 134 (44.82%) patients, while first-degree and second-degree obesity was diagnosed in 87 (29.10%). One hundred and seventy-nine (59.87%) participants admitted to receiving treatment for hypertension, 95 (31.77%) were treated for hypercholesterolemia, 70 (23.41%) had type 2 diabetes, and 133 (44.48%) met the IFD criteria for metabolic syndrome (MetS).

The frequency of the genotypes and alleles of the polymorphisms analyzed in our study are shown in Table 2. In each of the studied *loci*, it was in conformity with the Hardy–Weinberg principle (*SRD5A*: rs6884552, *p* = 0.0244; *SRD5A1*: rs523349, *p* = 0.0004 and rs12470143, *p* < 0.0001).

We analyzed the relationships between the incidence of particular diseases and the genotypes of the *SRD5A1* and *SRD5A2* polymorphisms among patients with BPH (Table 3). The BPH patients with the CC genotype of the *SRD5A2* rs523349 and rs12470143 polymorphisms were considerably less frequently diagnosed with MetS (*p* = 0.022 and *p* = 0.023 respectively). There were no significant relationships between the tested polymorphism genotypes and the incidence of health problems such as obesity, hypertension, type 2 diabetes, and hypercholesterolemia.

We also assessed the relationships between the genotypes and anthropometric, metabolic, and hormonal parameters (Table 4). Our analysis revealed that the homozygotes with the CC of the *SDR5A2* rs12470143 polymorphism had visibly higher HDL levels than those with the TT and CT genotypes (*p* = 0.001). Additionally, we found that the patients with the CC genotype of the *SDR5A2* rs12470143 polymorphism had considerably higher FT levels (*p* = 0.001) than the heterozygotes with the CT and the homozygotes with the TT of the genetic variant analyzed in our study. Furthermore, the patients with at least one G allele of the *SRD5A2* rs523349 polymorphism had significantly lower SGBG levels (*p* = 0.022) compared with the homozygotes with the CC genotype. The presence of at least one A allele (AA + AG genotypes) of the *SRD5A1* rs3797177 polymorphism entailed notably lower serum insulin levels than those observed in homozygotes with the GG genotype (*p* = 0.033). Logistic regression (Table 5) demonstrated that the *SRD5A2* (rs12470143) polymorphism contributed slightly to the HDL level (OR = 1.020; *p* = 0.010).

Analysis of the haplotypes of the *SDR5A1* and *SRD5A2* genes (Table 6) showed that there was no statistical relationship between the frequency of the *SRD5A1* haplotypes and the occurrence of MetS in the patients with BPH. Nevertheless, we noticed that the CC haplotype of the tested *SRD5A2* polymorphisms was significantly more common in the patients without MetS than in their counterparts with this health problem (*p* = 0.023).

## 4. Discussion

5α-reductase types 1 and 2, which are products of the *SRD5A1* and *SRD5A2* expression, are associated with the process of reducing testosterone to dihydrotestosterone. Since the prostate is a gland dependent on androgens, these genes may play a part in the etiology and treatment of both BPH and prostate tumors [8]. The relationship between the incidence of prostate diseases and the *SRD5A2* polymorphism has been confirmed by many studies [8,13,14]. Reports on the association between the *SRD5A1* polymorphism and BPH are less numerous [15].

The connections between polymorphic variants of the tested genes in BPH patients, and the levels of metabolic and hormonal parameters, have not yet been fully explained. Since the hypothesis about the etiology of BPH takes into account the levels of risk factors for cardiovascular disease, research on these issues seems crucial [16].

Our study revealed that the *SRD5A1* and *SRD5A2* genes can play a role in shaping the metabolic profiles of patients with BPH. It demonstrated that the *SRD5A2* rs12470143 polymorphism can influence the blood lipid profile. The CC genotype of the *SRD5A2* rs12470143 polymorphism protects against BPH, and involves higher serum HDL levels. Hazlehurst et al. [17] informed that therapy with 5α-reductase inhibitors may be related to the development of insulin resistance in liver cells, and the accumulation of lipids in the liver without affecting tissue sensitivity to peripheral insulin. The aforementioned authors provided evidence that the *SRD5A1* polymorphism can be involved in metabolic homeostasis. What is important is that both isoforms of 5α-reductase are found in liver cells [18], while only *SRD5A1* expression is observed in adipocytes [19]. In the animal model-based study conducted by Krawczyńska et al. [20], the strength of the *SRD5A1* expression correlated positively with the body weight of the tested Wistar rats receiving a high-fat diet.

Our study indicated the possibility of genetic linkage between the *SRD5A2* haplotypes, which could explain a higher MetS incidence among BPH patients. Although we did not notice any relationships between the tested polymorphisms and serum total testosterone levels, the CC genotype of the *SRD5A2* (rs12470143) polymorphism entailed increased levels of free testosterone in the BPH patients. As stated by Kristal et al. [21], higher TT levels do not enhance the risk of BPH. According to these authors, genetic factors decreasing the TT conversion to DHT can reduce the risk of clinical BPH symptoms.

Gu, X. et al. [15] reported on the connection between the *SRD5A1* (rs6884552 and rs3797177) and *SRD5A2* (rs523349) polymorphisms, and the effectiveness of BPH treatment with inhibitors of receptors for 5α-reductase type 2 and agents exerting an effect on α-adrenergic receptors. However, an aspect analyzed in their study was prostate gland volume, and the authors did not take into account the levels of metabolic and hormonal parameters in blood. Other researchers maintain that treating BPH with antagonists of 5α-reductase type 2 can diminish the risk of fast BPH development, even by 50% [22,23]. Combined therapy with both types of drugs (5α-reductase types 1 and 2) can reduce the risk by even 66% [24]. Azzouzi et al. [10] deny the contribution of the *SRD5A2* polymorphic variant (TA repeats, V89L and A49T mutations) to BPH, and even suggest its protective effect.

El Ezzi et al. [14] indicated the relationship between the *SDR5A2* polymorphic variant (R-5’GCCAGCTGGCAGAACGCCAGGAGAC3’) and a higher risk of prostate cancer. Other studies, among them the study of El Ezzi et al. [9], imply that the *SRD5A2* polymorphism (TA Repeats) may be directly associated with a higher risk of BPH in men. An interesting research study was carried out by Wang et al. [25] that informed about the possibility of a biochemical pathway converting androgens into estrogens when no *SRD5A2* expression is observed in prostate cells. They demonstrated that epigenetic silencing of this gene can have an impact on homeostasis and an increase in prostate volume.

## 5. Conclusions

The study described in this article shows an association between selected SRD5A1 and SRD5A2 polymorphisms and the levels of metabolic and hormonal parameters in patients with BPH. Special attention should be paid to the SDR5A2 rs12470143 polymorphism, which is associated with a change in lipid profile. Analysis of the frequency of this polymorphism among BPH patients could be useful in estimating the risk of getting ill, and planning therapies of concomitant diseases for BPH patients.

### Limitations of the Study 

The limitation of our study was the lack of a control group, i.e., men without BPH. Another drawback was the fact that blood pressure was measured only once in each patient, which was data that we did not allow for inclusion in the study. The unscreened control groups, such as patients with BPH cohorts, are frequently used in case-control genetic association studies. In our study, patients in the examined subgroups were ethnically and geographically matched, and regardless of inherent differences in some demographic characteristics between the groups, this study design is not likely a major limitation. Due to the preliminary character of the study, we did not use multiple testing corrections; therefore, the results should be interpreted with caution.

## Figures and Tables

**Table 1:** Characteristics of the study group (*n* = 299).

Variable	X	SD	Me	Min	Max
Age [year]	67.04	7.50	66.00	44.00	85.00
Weight [kg]	83.84	14.45	82.00	51.50	125.00
BMI [kg/m^2^]	28.03	4.38	27.51	19.15	40.90
WC [cm]	99.59	11.08	99.00	75.00	138.00
FPG [mg/dL]	95.61	15.65	94.50	58.00	138.50
BPS [mmHg]	119.91	13.76	120.00	90.00	160.00
BPD [mmHg]	77.42	9.84	80.00	55.00	90.00
HDL [mg/dL]	51.60	20.30	51.11	20.70	97.00
TG [mg/dL]	134.58	53.26	126.79	46.00	339.00
TC [mg/dL]	190.90	47.64	186.00	83.79	315.00
LDL [mg/dL]	113.13	47.99	106.29	24.40	287.19
TT [ng/mL]	4.19	1.97	4.04	0.09	11.13
FT free [pg/mL]	40.61	48.99	18.70	0.09	280.20
LH [mIU/mL]	9.69	6.67	8.13	0.70	67.98
SHBG [nmoL/L]	40.13	17.96	38.42	2.58	98.03
I [µlU/mL]	18.17	19.03	11.63	0.00	112.89
*Categorical variables*		*n*		%	
BMI <25 kg/m^2^		221		73.91	
BMI <30 kg/m^2^		87		29.10	
BMI <35 kg/m^2^		22		16.19	
Hypertension		179		59.87	
Diabetes		70		23.41	
*Hypercholesterolemia*		95		31.77	
MetS (IDF, 2005)		133		44.48	

X—average; SD—standard deviation; Me—median; Min—minimum, Max—maximum; BMI—body mass index; WC —abdominal circumference; FPG—fasting plasma glucose; BPS—Blood pressure systolic; BPD—blood pressure diastolic; HDL—high-density lipoprotein; TG—triglycerides; TC—total cholesterol; LDL—low-density lipoprotein; TT—total testosterone; FT—free testosterone; LH—luteinizing hormone; SHBG—sex hormone binding globulin; I—insulin; *n*—number; MetS–metabolic syndrome.

**Table 2:** Genotype and allele distribution in the study group (*n* = 299).

SNP	Genotype/Allele	Number of Genotypes/Alleles (299/598)	%	*p*
*SRD5A1* (rs6884552)	CC	136	(45.48)	0.0244 *
	CT	118	(39.46)	
	TT	45	(15.05)	
	C	390	(65.22)	
	T	208	(34.78)	
*SRD5A1* (rs3797177)	AA	208	(69.57)	0.935
	AG	83	(27.76)	
	GG	8	(2.68)	
	A	499	(83.44)	
	G	99	(16.56)	
*SRD5A2* (rs523349)	CC	157	(52.51)	0.0004 *
	CG	101	(33.78)	
	GG	41	(13.71)	
	C	415	(69.40)	
	G	183	(30.60)	
*SRD5A2* (rs12470143)	CC	133	(44.48)	<0.0001 *
	CT	102	(34.11)	
	TT	64	(21.40)	
	C	368	(61.54)	
	T	230	(38.46)	

SNP—single nucleotide polymorphism; A—adenine; T—thymine; C—cytosine; G—guanine; *n*—number; *p*—statistical significance; *—statically significant parameter.

**Table 3:** Diseases of patients with benign prostatic hyperplasia, *n* = 299.

Variable		*SRD5A1*						*SRD5A2*					
		rs6884552			rs3797177			rs523349			rs12470143		
		CC (*n* = 136)	TT + TC (*n* = 163)	*p*	GG (*n* = 8)	AA + AG (*n* = 291)	*p*	CC (*n* = 157)	GG + CG (*n* = 142)	*p*	CC (*n* = 133)	TT + CT (*n* = 166)	*p*
BMI	≥25	32 (10.7)	46 (15.4)	0.469	2 (0.7)	76 (25.4)	0.684	36 (12.0)	41 (13.7)	0.147	37 (12.4)	42 (14.0)	0.825
	25–29.99	67 (22.4)	67 (22.4)		5 (1.7)	129 (43.1)		78 (26.1)	56 (18.7)		56 (18.7)	79 (26.4)	
	30–34.99	28 (9.4)	37 (12.4)		1 (0.3)	64 (21.4)		35 (11.7)	30 (10.0)		30 (10.0)	34 (11.4)	
	≤35	9 (3.0)	13 (4.3)		0 (0.0)	22 (7.4)		8 (2.7)	14 (4.7)		10 (3.3)	11 (3.7)	
Diabetes	No	101 (33.8)	128 (42.8)	0.386	6 (2.0)	223 (74.6)	0.914	122 (40.8)	107 (35.8)	0.631	105 (35.1)	122 (40.8)	0.261
	Yes	35 (11.7)	35 (11.7)		2 (0.7)	68 (22.7)		35 (11.7)	35 (11.7)		28 (9.4)	44 (14.7)	
Hypertension	No	50 (16.7)	70 (23.4)	0.278	4 (1.3)	116 (38.8)	0.564	64 (21.4)	56 (18.7)	0.815	53 (17.7)	69 (23.1)	0.759
	Yes	86 (28.8)	93 (31.1)		4 (1.3)	175 (58.5)		93 (31.1)	86 (28.8)		80 (26.8)	97 (32.4)	
Hypercholesterolemia	No	100 (33.4)	104 (34.8)	0.072	5 (1.7)	199 (66.6)	0.724	107 (35.8)	97 (32.4)	0.977	90 (30.1)	113 (37.8)	0.948
	Yes	36 (12.0)	59 (19.7)		3 (1.0)	92 (30.8)		50 (16.7)	45 (15.1)		43 (14.4)	53 (17.7)	
MetS	No	71 (23.7)	95 (31.8)	0.292	3 (1.0)	163 (54.5)	0.299	97 (32.4)	69 (23.1)	0.022 *	83 (27.8)	82 (27.4)	0.023 *
	Yes	65 (21.7)	68 (22.7)		5 (1.7)	128 (42.8)		60 (20.1)	73 (24.4)		50 (16.7)	84 (28.1)	

BMI—body mass index; MetS—metabolic syndrome; A—adenine; T—thymine; C—cytosine; G—guanine; *n*—number; *p*—statistical significance; *—statically significant parameter.

**Table 4:** Relations between the anthropometric, metabolic, and hormonal parameters, and the *SRD5A1* (rs6884552 and rs3797177) and *SRD5A2* (rs523349 and rs12470143) gene polymorphisms.

Variable	*SRD5A1*						*SRD5A2*					
	rs6884552			rs3797177			rs523349			rs12470143		
	CC (*n* = 136)	TT + TC (*n* = 163)	*p*	GG (*n* = 8)	AA + AG (*n* = 291)	*p*	CC (*n* = 157)	GG + CG (*n* = 142)	*p*	CC (*n* = 133)	TT + CT (*n* = 166)	*p*
	X ± SD	X ± SD		X ± SD	X ± SD		X ± SD	X ± SD		X ± SD	X ± SD	
Weight [kg]	84.05 ± 13.2	83.66 ± 15.5	0.559	84.01 ± 14.5	77.75 ± 9.0	0.209	83.04 ± 12.8	84.72 ± 16.0	0.523	84.37 ± 15.1	83.18 ± 13.6	0.754
BMI [kg/m^2^]	28.05 ± 4.2	28.02 ± 4.6	0.772	28.07 ± 4.4	26.79 ± 2.9	0.403	27.98 ± 3.9	28.10 ± 4.8	0.732	28.16 ± 4.4	27.86 ± 4.2	0.801
WC [cm]	100.11 ± 11.07	99.16 ± 11.1	0.306	99.69 ± 11.2	96.00 ± 7.3	0.325	99.45 ± 10.2	99.75 ± 11.9	0.837	99.76 ± 12.1	99.49 ± 9.9	0.646
FPG [mg/dL]	95.08 ± 13.0	96.04 ± 17.5	0.937	95.39 ± 15.3	103.42 ± 25.0	0.352	95.26 ± 16.0	96.04 ± 15.2	0.788	94.81 ± 16.4	96.42 ± 14.2	0.495
HDL [mg/dL]	49.24 ± 20.9	53.55 ± 19.0	0.073	51.46 ± 20.4	57.28 ± 16.5	0.485	52.58 ± 20.5	50.54 ± 20.0	0.341	58.33 ± 19.6	45.81 ± 19.1	0.001 *
TG [mg/dL]	132.42 ± 49.5	136.37 ± 56.3	0.876	134.72 ± 53.02	128.75 ± 66.7	0.493	132.68 ± 53.1	136.64 ± 53.4	0.652	132.83 ± 53.4	136.20 ± 53.6	0.445
CT [mg/dL]	188.72 ± 47.8	192.69 ± 47.6	0.638	190.39 ± 47.6	211.67 ± 46.2	0.224	194.59 ± 47.5	186.94 ± 47.6	0.199	192.46 ± 49.2	189.34 ± 46.5	0.424
LDL [mg/dL]	112.92±48.2	113.30 ± 47.0	0.964	112.74 ± 47.9	128.68 ± 52.0	0.417	118.03 ± 51.0	107.80 ± 44.0	0.146	107.35 ± 46.7	117.79 ± 48.8	0.196
Hormonal Parameters												
TT [ng/mL]	4.11 ± 1.9	4.25 ± 2.0	0.669	4.16 ± 1.9	5.08 ± 2.5	0.394	4.27 ± 1.9	4.09 ± 2.0	0.352	4.22 ± 1.9	4.12 ± 1.9	0.761
FT [pg/mL]	41.46 ± 54.7	39.90 ± 43.9	0.591	40.67 ± 49.1	38.11 ± 43.9	0.948	42.28 ± 51.5	38.80 ± 46.1	0.755	50.54 ± 51.0	31.12 ± 42.9	0.001 *
LH [mIU/mL]	10.06 ± 6.0	9.37 ± 7.2	0.128	9.73 ± 6.7	8.04 ± 4.5	0.468	10.14 ± 7.0	9.18 ± 6.2	0.097	9.12 ± 6.1	10.20 ± 7.1	0.104
SHBG [nmol/L]	40.41 ± 17.6	39.90 ± 18.3	0.640	40.06 ± 17.7	43.39 ± 26.0	0.932	41.81± 16.7	38.32 ± 19.0	0.022 *	40.02 ± 17.4	40.18 ± 18.4	0.891
I [mIU/mL]	18.75 ± 21.3	17.72 ± 17.1	0.805	18.46 ± 19.1	5.59 ± 3.7	0.033 *	18.10 ± 20.3	18.26 ± 17.4	0.551	16.95 ± 16.2	19.12 ± 21.2	0.895

X—average; SD—standard deviation; BMI—body mass index; WC—abdominal circumference; FPG—fasting plasma glucose; HDL—high-density lipoprotein; TG—triglycerides; TC—total cholesterol; LDL—low-density lipoprotein; TT—total testosterone; FT—free testosterone; LH—luteinizing hormone; SHBG—sex hormone binding globulin; I—insulin; A—adenine; T—thymine; C—cytosine; G—guanine; *n*—number; *p*—statistical significance; *—statically significant parameter.

**Table 5:** The influence of recessive *SRD5A2* (rs12470143): CC vs. TT and CT genotype on metabolic and anthropometric indices by means of logistic regression.

Variable	*p*	OR	CI < 95%	CI > 95%
WC [cm]	0.765	1.004	0.976	1.034
FPG [mg/dL]	0.913	0.999	0.980	1.018
HDL [mg/dL]	0.010 *	1.020	1.005	1.035
TG [mg/dL]	0.802	0.999	0.994	1.005

OR—odds ratio; CI—confidence interval; A—adenine; T—thymine; C—cytosine; G—guanine; WC—waist circumference; HDL—high-density lipoprotein; TG—triglyceride; FPG—fasting plasma glucose; *p*—statistical significance; *—statically significant parameter.

**Table 6:** The haplotype frequency of the *SRD5A1* (rs6884552 and rs3797177) and *SRD5A2* (rs523349 and rs12470143) polymorphisms in benign prostatic hyperplasia (BPH) patients with and without MetS.

*SRD5A1* (rs6884552 i rs3797177)					
Haplotype	Hap.Score	*p*	Total Frequency	Group of Patients without MetS	Group of Patients with MetS
T-G	−1.547	0.122	0.107	0.107	0.071
C-G	0.852	0.394	0.074	0.080	0.066
T-A	0.098	0.922	0.248	0.246	0.251
C-A	1.144	0.252	0.587	0.567	0.613
*SRD5A2* (rs523349 i rs12470143)					
C-C	−2.27304	0.023 *	0.345	0.392	0.284
G-T	−0.09986	0.920	0.033	0.036	0.026
C-T	1.01812	0.309	0.352	0.329	0.383
G-C	1.44430	0.149	0.270	0.243	0.307

A—adenine; T—thymine; C—cytosine; G—guanine; *n*—number; *p*—statistical significance; *—statically significant parameter.
